# Association Between the COGAS Score and Delayed Neuropsychiatric Sequelae After Acute Carbon Monoxide Poisoning

**DOI:** 10.3390/jcm15114322

**Published:** 2026-06-03

**Authors:** Sun Chul Lee, Gyo Jin Ahn

**Affiliations:** 1 Department of Emergency Medicine, Yonsei University Wonju College of Medicine, Wonju 26426, Republic of Korea; emsclee@yonsei.ac.kr; 2 Research Institute of Hyperbaric Medicine and Science, Wonju Severance Christian Hospital, Yonsei University Wonju College of Medicine, Wonju 26426, Republic of Korea; 3 Research Institute of Resuscitation Science, Yonsei University Wonju College of Medicine, Wonju 26426, Republic of Korea

**Keywords:** carbon monoxide poisoning, delayed neuropsychiatric sequelae, COGAS: prognosis

## Abstract

**Background/Objectives**: Carbon monoxide (CO) poisoning remains a major cause of poisoning-related morbidity, and delayed neuropsychiatric sequelae (DNS) are important long-term complications. This study evaluated the association between the COGAS score and operationally defined 6-month DNS after acute CO poisoning. **Methods**: Overall, 272 patients with acute CO poisoning were included in this single-center observational cohort study. The primary outcome was operationally defined as 6-month DNS based on changes in the Global Deterioration Scale (GDS). Logistic regression analyses were performed to evaluate the association between the COGAS score and operationally defined 6-month DNS. Conventional and Firth penalized logistic regression models were used. **Results**: Among 272 patients, 14 (5.1%) met the criteria for operationally defined 6-month DNS. Each 1-point increase in the COGAS score was associated with higher odds of operationally defined 6-month DNS in univariable analysis (odds ratio [OR], 2.16; 95% confidence interval [CI], 1.30–3.65; *p* = 0.003) and in the fully adjusted model including initial GDS, troponin I, and CO exposure duration (OR, 2.19; 95% CI, 1.14–4.35; *p* = 0.020). **Conclusions**: Higher COGAS scores were associated with operationally defined 6-month DNS after acute CO poisoning. The COGAS score may provide prognostic information, but further validation in larger cohorts is needed.

## 1. Introduction

Carbon monoxide (CO) poisoning remains a major public health problem worldwide and continues to impose a substantial clinical burden [1,2,3]. In the United States alone, unintentional non-fire-related CO poisoning is associated with more than 400 deaths, 100,000 emergency department visits, and 14,000 hospitalizations annually [1,2]. Among its complications, neurological injury is one of the most clinically important because it can lead to persistent functional impairment even after apparent initial recovery [4,5]. In particular, delayed neuropsychiatric sequelae (DNS) are characterized by the delayed emergence of cognitive, behavioral, or motor dysfunction after a lucid interval and represent a major cause of long-term morbidity after acute CO poisoning [4,6,7]. A major challenge in the clinical management of CO poisoning is the inability to reliably predict DNS during the acute phase [8,9]. This complicates treatment decisions and follow-up planning, because some patients who initially appear neurologically stable later develop clinically meaningful deterioration, whereas others recover without delayed complications.

Several predictors of neurologic outcomes after CO poisoning have been proposed, including a low Glasgow Coma Scale (GCS) score, elevated creatine kinase, serum neuron-specific enolase, abnormal early neuroimaging findings, and other routinely available clinical or biochemical variables such as blood urea nitrogen [10,11,12,13,14,15,16,17,18,19,20]. However, many of these approaches have important limitations, including restricted accessibility, delayed availability, high cost, and incomplete standardization across institutions [21]. In addition, some markers may not be readily available during initial evaluation, which may limit their usefulness for immediate bedside risk stratification. Although hyperbaric oxygen therapy (HBOT) may still be considered in selected patients with suspected neurological involvement, no clearly established treatment exists once DNS develops [8,22,23]. Accordingly, the early identification of patients at high risk of delayed deterioration is clinically important.

Therefore, a simple and clinically applicable bedside tool is required for early risk stratification. The COGAS was originally developed to predict poor neurocognitive outcomes after acute CO poisoning using readily available clinical variables [24]. However, its utility in predicting long-term delayed neurological deterioration has not been sufficiently evaluated. In particular, whether the COGAS score can be applied to risk stratification for clinically meaningful DNS at 6 months remains unclear.

To address these gaps, in this study, we investigated the association between COGAS scores and 6-month DNS after acute CO poisoning. Unlike the original derivation study, which focused on short-term poor neurocognitive outcomes, the present study focused on longer-term delayed neuropsychiatric deterioration. We analyzed the score as both a continuous variable and according to predefined cutoff values to examine whether higher COGAS scores were associated with a greater risk of 6-month DNS.

## 2. Materials and Methods

### 2.1. Study Design and Setting

This observational cohort study was performed at a tertiary academic hospital serving as a regional emergency medical center in Korea. The emergency department has 34 beds and receives more than 46,000 visits annually. A prospective registry for patients with acute CO poisoning has been maintained at our institution since January 2006. Patient data collected between 8 January 2006 and 31 July 2020 were prospectively recorded in the institutional registry and retrospectively analyzed. Since 1 August 2020, data collection has continued prospectively with informed consent as part of the Carbon Monoxide Intoxication in Korea: Prospective Cohort (CARE CO cohort; ClinicalTrials.gov identifier: NCT04490317). For the present 6-month DNS analysis, the registry was screened for patients with CO poisoning who presented to the ED between 8 January 2006 and 31 January 2026. The data were locked on 31 January 2026. Because the primary outcome was assessed at 6 months, patients who presented after 31 July 2025 were considered not yet eligible for 6-month outcome assessment and were excluded from the analytic cohort. This study was approved by the hospital’s Institutional Review Board (IRB No. CR326010). The requirement for informed consent was waived for data collected before August 2020 owing to the retrospective analysis of prospectively collected registry data. For subsequent data collection, written informed consent was obtained from patients or their legal guardians. All procedures were conducted in accordance with the principles of the Declaration of Helsinki. All patient data were anonymized before analysis.

### 2.2. Study Population, Variables, and Definition

Consecutive patients with CO poisoning who visited the ED were screened for eligibility. Acute CO poisoning was defined by clinical features consistent with CO exposure, a recent exposure history, and elevated carboxyhemoglobin (CO-Hb) levels, using thresholds of >5% for non-smokers and >10% for smokers. Clinical variables were selected based on known risk factors for CO poisoning and the components of the COGAS score. These variables included age, sex, intentionality of poisoning, CO source, drug co-ingestion, GCS score at rescue or ED arrival, comorbidities, alcohol co-ingestion, current smoking status, symptoms and signs at ED presentation, HBOT treatment, CO exposure duration, laboratory findings, GDS assessments, and the COGAS score. CO source was categorized as charcoal, firewood, gas, or fire. Comorbidities included diabetes mellitus, hypertension, cardiovascular disease, and psychiatric disease. Symptoms and signs at ED presentation included loss of consciousness, shock, and seizure. Laboratory findings included CO-Hb, bicarbonate, lactate, creatinine, creatine kinase, and troponin I levels measured within 1 h of ED arrival. GDS assessments included initial, 1-month, and 6-month GDS scores. Shock was defined as requiring vasopressor support and having a serum lactate level > 2 mmol/L. CO exposure duration was defined as the estimated interval from the last known alert or exposure time to patient discovery. Patients were excluded sequentially according to the following criteria: 6-month follow-up not yet due at the data lock date; age < 16 years; non-acute CO poisoning; unknown or documented previous CO poisoning history; previous stroke; previous neurocognitive disease; previous or unknown history of cancer; receipt of specific additive treatments, including therapeutic hypothermia or steroid therapy; unavailable variables required for COGAS calculation; absence of an initial Global Deterioration Scale (GDS) assessment; or absence of a 6-month GDS assessment.

### 2.3. COGAS Score and Outcome Measure

The COGAS score, developed in a previous study, was used to evaluate the risk of neurocognitive deterioration [24]. The score consists of five predefined variables, each assigned 1 point: age > 50 years, GCS score ≤ 12, presence of shock, absence of HBOT, and creatine kinase level > 320 U/L. The total score ranged from 0 to 5, with higher scores indicating greater risk. All variables included in the COGAS were assessed at the initial presentation. Other collected variables were excluded from the score calculation to preserve the original model structure.

Neurocognitive outcomes were assessed using the GDS, which ranges from 1 to 7, with higher scores indicating more severe cognitive impairment (Table S1). During the acute phase, the initial GDS score was assessed in hospitalized patients by a rehabilitation medicine specialist through formal consultation after clinical stabilization, rather than at the time of ED arrival, to minimize the potential influence of acute CO intoxication, alcohol co-ingestion, or drug co-ingestion on cognitive assessment. Hospital admission was generally recommended for patients with laboratory abnormalities, abnormal imaging findings, or persistent symptoms such as dizziness or headache despite HBOT. However, patients who did not wish to be hospitalized were discharged from the ED and therefore did not undergo the inpatient initial GDS assessment. Follow-up GDS evaluations at 4–6 weeks and 6 months after CO exposure were primarily performed through reassessment by a rehabilitation medicine specialist. When outpatient reassessment was not feasible, GDS status was determined, when possible, through structured telephone interviews with caregivers conducted by trained personnel.

The primary outcome was operationally defined 6-month DNS. Because no universally accepted diagnostic criteria exist for DNS after CO poisoning and definitions vary across studies, 6-month DNS was defined in this study using longitudinal changes in GDS scores from the initial assessment to the 6-month follow-up. Patients were classified as having operationally defined 6-month DNS if they met either of the following criteria: (1) an initial GDS score of 1 to 3 and a 6-month GDS score of 4 to 7 with an increase of at least 1 point, or (2) an initial GDS score of 4 or higher with further worsening by at least 1 point at 6 months. This definition was intended to capture clinically meaningful delayed neurocognitive or functional deterioration while accounting for baseline impairment during the acute phase. Sensitivity analyses were performed using alternative outcome definitions reflecting classical DNS-like deterioration and further deterioration among patients with baseline impairment.

### 2.4. Statistical Analysis

Categorical variables are presented as frequencies and percentages and were compared using Pearson’s chi-square test or Fisher’s exact test, as appropriate. Continuous variables were assessed for normality using the Shapiro–Wilk test and are presented as medians with interquartile ranges (IQRs). Comparisons between groups were performed using the Mann–Whitney U test.

The association between the COGAS score and operationally defined 6-month DNS was evaluated using logistic regression. For the continuous COGAS model, effect estimates were reported as odds ratios (ORs) with 95% confidence intervals (CIs) per 1-point increment in the score. Univariable and multivariable models were constructed using prespecified covariate sets, including initial GDS, CO exposure duration, troponin I, and combinations of these variables. The model adjusted for all three covariates was considered the fully adjusted model.

Predefined COGAS cutoffs of ≥2, ≥3, and ≥4 were evaluated as exploratory risk-stratification thresholds. For each cutoff, ORs with 95% CIs were estimated using logistic regression. The incidence of operationally defined 6-month DNS was also calculated according to the exact COGAS score to describe the distribution of outcome events across score categories.

To reduce small-sample and sparse-data bias, Firth penalized logistic regression was performed alongside conventional logistic regression for the main continuous-score models, predefined cutoff models, and sensitivity analyses.

Discrimination and classification performance were evaluated descriptively. Receiver operating characteristic curve analysis and precision-recall curve analysis were performed, and the area under the receiver operating characteristic curve (ROC-AUC) and area under the precision-recall curve (PR-AUC) were calculated for the COGAS score. For predefined cutoffs of ≥2, ≥3, and ≥4, sensitivity, specificity, positive predictive value, and negative predictive value were calculated. Because of the small number of outcome events, these predictive performance analyses were considered exploratory, and formal calibration assessment was not performed.

Sensitivity analyses were performed to evaluate the robustness of the findings. First, the association between the COGAS score and alternative outcome definitions, including classical DNS-like deterioration and further deterioration among patients with baseline impairment, was examined. Second, a component-specific sensitivity analysis was performed using a 4-component COGAS score that excluded the HBOT item.

A two-sided *p*-value threshold of <0.05 was used to define statistical significance. All analyses were conducted using R software, version 4.5.3 (R Foundation for Statistical Computing, Vienna, Austria).

## 3. Results

### 3.1. Characteristics of the Study Population

**Figure 1:**
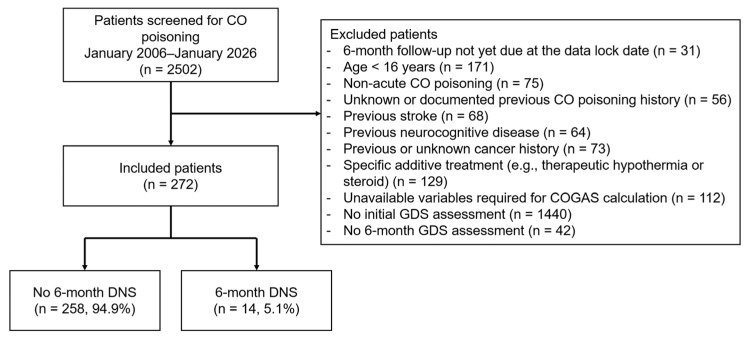
Flow diagram of patient selection for the operationally defined 6-month delayed neuropsychiatric sequelae analysis. CO, carbon monoxide; DNS, delayed neuropsychiatric sequelae; GDS, Global Deterioration Scale.

After sequential application of the eligibility criteria, 272 patients were included in the final analytic cohort (Figure 1). Among them, 14 patients (5.1%) met the criteria for operationally defined 6-month DNS, whereas 258 patients (94.9%) did not. Among the 42 patients excluded because of the absence of a 6-month GDS assessment, one died between the 1-month and 6-month follow-up evaluations, and 41 were lost to follow-up or declined further contact. The baseline characteristics of the analytic cohort according to 6-month DNS status are summarized in Table 1.

Most baseline demographic and clinical variables did not differ significantly between patients with and without operationally defined 6-month DNS. Patients with 6-month DNS were older and had lower initial GCS scores, although these differences were not statistically significant (*p* = 0.126 and *p* = 0.066, respectively). Creatine kinase, troponin I, and COGAS scores were significantly higher in patients with 6-month DNS than in those without 6-month DNS (*p* = 0.016, *p* = 0.001, and *p* = 0.005, respectively). Initial GDS scores were similar between the groups (*p* = 0.136). However, patients with 6-month DNS had higher GDS scores at both 1 month and 6 months than those without 6-month DNS (both *p* < 0.001).

Baseline characteristics according to the availability of the initial GDS assessment are presented in Table S2, and the distribution of COGAS score categories according to initial GDS availability is shown in Table S3. Compared with patients without initial GDS assessment, those with available initial GDS assessment were older (*p* = 0.013), were more frequently treated with HBOT (*p* < 0.001), had longer CO exposure duration (*p* = 0.006), and had higher CO-Hb, creatine kinase, and troponin I levels (*p* < 0.001, *p* = 0.024, and *p* < 0.001, respectively). Patients with and without 6-month DNS showed similar median COGAS scores (*p* = 0.912).

### 3.2. Association Between the COGAS Score and Operationally Defined 6-Month DNS

Higher COGAS scores were associated with operationally defined 6-month DNS (Table 2). In univariable logistic regression analysis, each 1-point increase in the COGAS score was associated with higher odds of operationally defined 6-month DNS in both conventional logistic regression and Firth penalized logistic regression (conventional: OR, 2.16; 95% CI, 1.30–3.65; *p* = 0.003; Firth: OR, 2.13; 95% CI, 1.30–3.55; *p* = 0.003). In the fully adjusted model, the association remained statistically significant in both conventional logistic regression and Firth penalized logistic regression (conventional: OR, 2.19; 95% CI, 1.14–4.35; *p* = 0.020; Firth: OR, 2.09; 95% CI, 1.11–4.06; *p* = 0.022).

**Figure 2:**
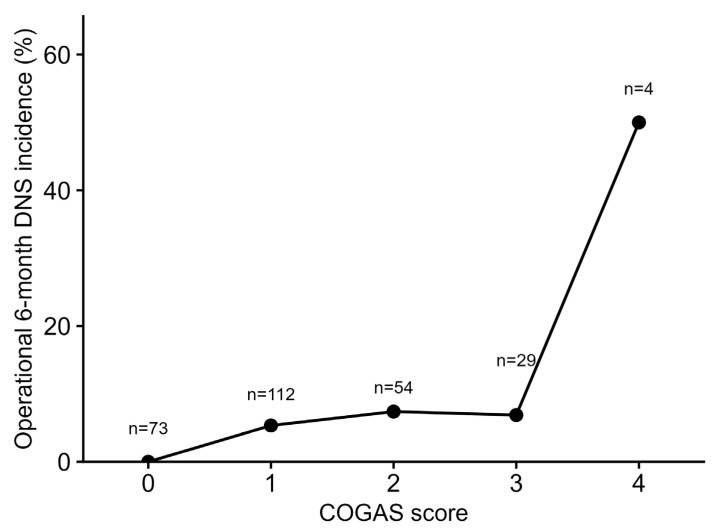
Incidence of operationally defined 6-month delayed neuropsychiatric sequelae by COGAS score. Numbers above each point indicate the number of patients in each COGAS score category.

The incidence of operationally defined 6-month DNS according to the COGAS score was 0%, 5.4%, 7.4%, 6.9%, and 50.0% for scores of 0, 1, 2, 3, and 4, respectively (Figure 2). The corresponding numbers of events and patients were 0/73, 6/112, 4/54, 2/29, and 2/4, respectively.

Sensitivity analyses using alternative outcome definitions are presented in Table S4. For classical DNS-like deterioration, the association between the COGAS score was not statistically significant in the unadjusted model but was statistically significant after adjustment in both conventional and Firth penalized logistic regression models (conventional: adjusted OR, 2.51; 95% CI, 1.11–5.70; *p* = 0.024; Firth: adjusted OR, 2.39; 95% CI, 1.08–5.23; *p* = 0.032). For progressive deterioration, the unadjusted association was statistically significant (conventional: OR, 3.61; 95% CI, 1.55–10.18; *p* = 0.006; Firth: OR, 3.33; 95% CI, 1.50–8.69; *p* = 0.003), whereas the adjusted association was not statistically significant.

Sensitivity analyses of DNS progression from 1 to 6 months are presented in Table S5. For operationally defined 6-month DNS progression from 1 to 6 months, the unadjusted association was statistically significant in both conventional and Firth penalized logistic regression models (conventional: OR, 2.45; 95% CI, 1.09–5.97; *p* = 0.033; Firth: OR, 2.37; 95% CI, 1.10–5.46; *p* = 0.029), whereas the adjusted association did not reach statistical significance. In the component-specific sensitivity analysis using the 4-component COGAS score excluding the HBOT item, the unadjusted association was statistically significant in both models, whereas the adjusted association did not reach statistical significance (conventional: adjusted OR, 2.10; 95% CI, 0.91–4.98; *p* = 0.083; Firth: adjusted OR, 2.02; 95% CI, 0.91–4.66; *p* = 0.086) (Table S6).

### 3.3. Exploratory Analyses of Predefined COGAS Cutoffs

Predefined COGAS cutoffs of ≥2, ≥3, and ≥4 were evaluated as exploratory cutoff-based thresholds for operationally defined 6-month DNS (Table 3). In the unadjusted cutoff analyses using conventional logistic regression, COGAS ≥ 2, ≥3, and ≥4 showed ORs of 3.02 (95% CI, 1.01–9.00), 3.16 (95% CI, 0.93–10.72), and 21.33 (95% CI, 2.76–164.67), respectively. The associations for the ≥2 and ≥4 cutoffs were statistically significant in the unadjusted analyses, whereas the association for the ≥3 cutoff did not reach statistical significance. In the fully adjusted conventional model, the corresponding ORs were 2.37 (95% CI, 0.61–9.24), 2.02 (95% CI, 0.45–9.16), and 11.19 (95% CI, 1.03–122.09), respectively. The ≥4 cutoff was the only threshold that remained statistically significant in the fully adjusted conventional model.

The classification characteristics varied across cutoffs. Sensitivity decreased from 57.1% at COGAS ≥ 2 to 28.6% at COGAS ≥ 3 and 14.3% at COGAS ≥ 4, whereas specificity increased from 69.4% to 88.8% and 99.2%, respectively. The positive predictive values were 9.2%, 12.1%, and 50.0%, and the negative predictive values were 96.8%, 95.8%, and 95.5% for COGAS ≥ 2, ≥3, and ≥4, respectively.

Associations between predefined COGAS cutoffs and operationally defined 6-month DNS across different adjustment models, including Firth penalized sensitivity analyses, are presented in Table S7. Across conventional and Firth models, the ≥4 cutoff generally showed the largest effect estimates and was statistically significant in most adjustment models. In the fully adjusted Firth model, the ≥4 cutoff showed an OR of 9.38 (95% CI, 0.93–80.36; *p* = 0.057). Discrimination performance is shown in Figure S1. The ROC-AUC of the COGAS score was 0.712 (95% CI, 0.596–0.828), and the PR-AUC was 0.162.

## 4. Discussion

This study showed that higher COGAS scores were associated with operationally defined 6-month DNS after acute CO poisoning. This association remained significant after adjustment for initial GDS, troponin I, and CO exposure duration, and Firth penalized logistic regression analyses yielded similar results for the continuous COGAS score. These findings suggest that the COGAS score may capture clinical information related to delayed neurocognitive deterioration beyond its original 1-month outcome framework. However, the cutoff-based analyses yielded wide confidence intervals because of the limited number of outcome events.

An important feature of this study is that the COGAS score was applied to a clinical outcome different from that used in the original derivation study. The original COGAS study developed the score to predict poor neurocognitive outcome at 1 month after acute CO poisoning, using GDS 1–3 as a favorable outcome and GDS 4–7 as a poor outcome [24]. In contrast, the present study focused on 6-month deterioration and applied an operational definition intended to capture delayed neurocognitive worsening over time.

Although the GDS was originally developed as a staging instrument for primary degenerative dementia [25], it has been used in previous CO poisoning studies as a pragmatic measure of global neurocognitive outcome. In addition to the original COGAS derivation study, a study of adjunctive therapeutic hypothermia combined with HBOT for acute severe CO poisoning used GDS assessments at both 1 and 6 months to evaluate longitudinal neurocognitive outcomes [26]. Therefore, the GDS was selected in the present study to maintain consistency with prior CO poisoning outcome research and the original COGAS framework while enabling longitudinal assessment of global cognitive and functional status. The GDS was not used to diagnose Alzheimer-type, vascular, or other degenerative dementia.

DNS has traditionally been described as the emergence of new neuropsychiatric symptoms or signs after apparent recovery or a lucid interval [4,8]. However, outcome definitions in CO poisoning studies have varied substantially. Some studies have focused on classical DNS-like deterioration, defined by newly developed neurological or psychiatric abnormalities after acute recovery, whereas others have evaluated broader delayed neurocognitive outcomes or longitudinal changes in global cognitive and functional status [22,24,26,27,28,29]. Accordingly, the present GDS-based outcome should be interpreted as an operationally defined delayed neurocognitive deterioration outcome rather than as a universally established or adjudicated DNS definition.

The two-component definition used in this study was intended to reflect two related but distinct outcome concepts. Classical DNS-like deterioration was used to capture patients who had no or only mild impairment at baseline but developed more clearly identifiable neuropsychiatric deterioration by 6 months. Progressive deterioration was used to capture additional worsening among patients who already had moderate or greater impairment at baseline. This component was not intended to classify persistent neurologic impairment after the acute injury as DNS. Rather, it required a further increase in GDS at 6 months, thereby distinguishing longitudinal worsening from persistent neurological sequelae without documented progression. In this subgroup, further GDS worsening was considered clinically relevant because higher GDS stages reflect greater global cognitive and functional impairment, although this trajectory is not equivalent to classical incident DNS after a lucid interval.

The composite primary outcome was therefore designed as a pragmatic approach to capture both incident delayed deterioration and longitudinal worsening. However, because the primary definition used initial and 6-month GDS assessments, it could not directly verify a lucid interval. In addition, changes within the lower GDS range may be more susceptible to variability in clinical assessment and subjective reporting. To address the possibility that milder delayed worsening may have been missed by the primary definition and to more closely examine delayed progression after the early post-exposure period, sensitivity analyses evaluating DNS progression from 1 to 6 months were additionally performed.

Sensitivity analyses using alternative outcome definitions provided additional context for the primary findings. The association between the COGAS score and classical DNS-like deterioration became statistically significant after adjustment, whereas the association with progressive deterioration was attenuated after adjustment. In the analysis of DNS progression from 1 to 6 months, the direction of association was generally similar, but the estimates were less precise. These findings suggest that the observed association was not restricted to a single outcome definition, but they also highlight the instability that arises when the outcome is divided into smaller component groups with few events.

Previous studies have suggested several potential predictors of delayed neurological sequelae after CO poisoning, including low initial GCS, elevated creatine kinase, serum neuron-specific enolase, abnormal early brain MRI findings, and other routinely available variables such as blood urea nitrogen [10,11,12,13,14,15,16]. However, each of these predictors has important limitations when considered separately. Reported GCS thresholds have varied across studies; creatine kinase may reflect systemic ischemia or muscle injury rather than delayed neurological injury itself; and neuron-specific enolase has shown heterogeneity in assay methods, measurement timing, and cutoff values [10]. Early brain MRI may also be helpful; however, its routine applicability is limited by restricted availability, heterogeneous imaging timing, incomplete standardization across studies, and practical difficulties in critically ill or clinically unstable patients [14,15]. In addition, some previously reported predictors were associated with broader poor outcomes rather than with DNS specifically [16]. Taken together, these limitations support the need for a structured clinical approach to early risk assessment.

In the present study, the incidence of operationally defined 6-month DNS was highest among patients with a COGAS score of 4. However, the incidence did not increase monotonically across all adjacent score categories, particularly between COGAS scores of 2 and 3. This non-monotonic pattern may reflect the small number of outcome events within individual COGAS score categories. Therefore, the score-category findings should be interpreted as descriptive rather than as evidence of a strictly stepwise dose–response relationship.

The predefined cutoff analyses provided additional exploratory information. Across conventional and Firth models, the ≥4 cutoff generally showed the largest effect estimates and was statistically significant in most adjustment models. However, the confidence intervals were wide, particularly for the ≥4 cutoff, reflecting the limited number of outcome events and sparse data. In the fully adjusted Firth model, the ≥4 cutoff showed a similar direction and magnitude of association, but the association did not reach conventional statistical significance. Therefore, the cutoff-based findings should be considered exploratory rather than confirmatory and require validation in larger cohorts with systematic neurologic assessment and follow-up.

The discrimination analysis also supports a cautious interpretation. The ROC-AUC suggested moderate discrimination, whereas the PR-AUC was low, which is expected in part given the low event rate of operationally defined 6-month DNS. Therefore, the present data support an association between the COGAS score and the outcome, but they do not establish the score as a validated prediction tool for clinical decision-making.

An additional issue relates to the HBOT component of the original COGAS score. Because most patients in the present cohort received HBOT, the “no HBOT” item showed limited variability and may have contributed little to discrimination in this dataset. To address this concern, a component-specific sensitivity analysis was performed using a 4-component COGAS score excluding the HBOT item. In this analysis, the association between the 4-component COGAS score and operationally defined 6-month DNS remained in the same direction, although the fully adjusted estimates were attenuated and did not reach statistical significance. This finding suggests that the overall direction of the association was not solely driven by the HBOT item. Nevertheless, HBOT is a time-dependent treatment, and treatment timing, number of sessions, and clinical indication were not incorporated into the binary HBOT component used in the original COGAS score. Therefore, the HBOT component should be interpreted cautiously, particularly in cohorts where HBOT is administered to most patients.

The COGAS score may be useful as a simple summary of routinely available clinical variables associated with delayed neurocognitive deterioration. However, the present study did not evaluate whether COGAS-guided follow-up or treatment decisions improve clinical outcomes. Incorporating objective neurologic assessments, neuropsychological testing, or neuroimaging findings may be considered in future validation studies.

This study had several limitations. First, due to its retrospective observational design, unmeasured confounding factors and hidden biases could not be excluded. Second, this study was conducted at a single center and included only Korean patients, which may limit the generalizability of the findings to other populations and clinical settings. Third, a substantial number of patients were excluded during cohort selection, including those without initial or follow-up GDS assessment. Patients with available initial GDS assessment differed from those without initial GDS assessment in several baseline characteristics, suggesting that the analytic cohort may have been enriched for patients with more clinically apparent or severe poisoning. Although the median COGAS score and the distribution of predefined COGAS score categories were similar according to initial GDS availability, residual selection bias remains possible. Fourth, the number of patients with operationally defined 6-month DNS was relatively small, which limited statistical power and contributed to wide confidence intervals in several analyses, particularly component-specific and cutoff-based analyses. Fifth, the HBOT component had limited variability and did not incorporate treatment timing, number of sessions, or clinical indication; moreover, this study was not designed to evaluate the treatment effect of HBOT, and no causal inference regarding HBOT efficacy can be made from these data. Sixth, the definition of DNS used in this study was an operational definition intended to capture clinically meaningful delayed neurocognitive deterioration and should not be interpreted as a universally established standard. Finally, although discrimination metrics were added, this study did not include formal calibration assessment, decision-curve analysis, or external validation. Because the number of outcome events was small, the added discrimination and classification metrics should be interpreted as exploratory. Further multicenter studies with larger cohorts, external validation, and systematic neurologic follow-up are needed to define the predictive performance and clinical utility of the COGAS score more precisely.

## 5. Conclusions

Higher COGAS scores were associated with operationally defined 6-month DNS after acute CO poisoning, even after adjustment for initial GDS, troponin I, and CO exposure duration. Sensitivity analyses showed generally consistent directions of association, although cutoff-based estimates were imprecise because of the limited number of outcome events. These findings suggest that the COGAS score may provide prognostic information regarding delayed neurocognitive deterioration, but they should be considered exploratory and require further validation in larger cohorts with systematic neurologic assessment and follow-up.

## Figures and Tables

**Table 1:** Baseline characteristics according to operationally defined 6-month delayed neuropsychiatric sequelae status.

	No 6-Month DNS (n = 258, 94.9%)	6-Month DNS (n = 14, 5.1%)	*p*-Value
Age	52 (36–62)	58.5 (47–68)	0.126
Sex			0.403
Woman	97 (37.6)	7 (50.0)	
Man	161 (62.4)	7 (50.0)	
Intentionality	97 (37.6)	6 (42.9)	0.779
CO source			0.948
Charcoal	187 (72.5)	10 (71.4)	
Firewood	14 (5.4)	1 (7.1)	
Gas	38 (14.7)	2 (14.3)	
Fire	19 (7.4)	1 (7.1)	
Drug co-ingestion	15 (5.8)	1 (7.1)	0.581
GCS score	15 (12–15)	12 (9–15)	0.066
Comorbidities			
Diabetes mellitus	31 (12.0)	3 (21.4)	0.395
Hypertension	57 (22.1)	5 (35.7)	0.322
Cardiovascular disease	11 (4.3)	0 (0.0)	1.000
Psychiatric disease	26 (10.1)	1 (7.1)	1.000
Alcohol co-ingestion	18 (7.0)	2 (14.3)	0.275
Current smoker	108 (41.9)	5 (35.7)	0.784
Symptoms and signs at ED presentation			
Loss of consciousness	135 (52.3)	10 (71.4)	0.182
Shock	4 (1.6)	1 (7.1)	0.234
Seizure	4 (1.6)	0 (0.0)	1.000
HBOT treatment	252 (97.7)	13 (92.9)	0.312
CO exposure duration (hours)	4.00 (1.31–8.00)	8.00 (3.00–9.00)	0.140
Laboratory findings			
CO-Hb (%)	22.50 (11.43–32.15)	26.85 (15.55–38.65)	0.326
Bicarbonate (mmol/L)	21.00 (19.00–22.70)	19.65 (17.60–21.10)	0.158
Lactate (mmol/L)	2.07 (1.26–3.30)	1.84 (1.28–2.91)	0.800
Creatinine (mg/dL)	0.81 (0.66–0.97)	0.86 (0.67–1.25)	0.344
Creatine kinase (U/L)	136.50 (90.00–294.50)	883.50 (115.50–5389.75)	0.016
Troponin I (pg/mL)	15.00 (15.00–138.43)	189.00 (59.00–2232.50)	0.001
Initial GDS	2 (1–3)	3 (1.25–4)	0.136
1-month GDS	1 (1–2)	6 (4.25–6.75)	<0.001
6-month GDS	1 (1–2)	6 (4.25–7)	<0.001
COGAS score	1 (0–2)	2 (1–3)	0.005

Note. Data are presented as n (%) or median (interquartile range), as appropriate. Troponin I values were harmonized to pg/mL; low values, including values reported as 0 or 15 pg/mL, reflect institutional reporting conventions during the study period. CO, carbon monoxide; CO-Hb, carboxyhemoglobin; ED, emergency department; GCS, Glasgow Coma Scale; GDS, Global Deterioration Scale; HBOT, hyperbaric oxygen therapy.

**Table 2:** Logistic regression models for operationally defined 6-month delayed neuropsychiatric sequelae according to the COGAS score.

Model	N	Events	Conventional	*p*-Value	Firth	*p*-Value
Univariable	272	14	2.16 (1.30–3.65)	0.003	2.13 (1.30–3.55)	0.003
Adjusted model 1	272	14	2.37 (1.28–4.47)	0.006	2.31 (1.27–4.28)	0.006
Adjusted model 2	272	14	2.16 (1.14–4.17)	0.019	2.08 (1.12–3.96)	0.020
Adjusted model 3	272	14	2.41 (1.27–4.68)	0.007	2.33 (1.25–4.42)	0.008
Adjusted model 4	272	14	2.19 (1.14–4.35)	0.020	2.09 (1.11–4.06)	0.022

Note. Values in the Conventional and Firth columns are ORs with 95% CIs per 1-point increase in the COGAS score. Conventional denotes conventional logistic regression, and Firth denotes Firth penalized logistic regression. Model 1 was adjusted for initial GDS; model 2 for initial GDS and troponin I; model 3 for initial GDS and CO exposure duration; and model 4 for initial GDS, troponin I, and CO exposure duration. Model 4 was considered the fully adjusted model. COGAS, carbon monoxide-associated global assessment score; CO, carbon monoxide; DNS, delayed neuropsychiatric sequelae; GDS, Global Deterioration Scale; OR, odds ratio; CI, confidence interval.

**Table 3:** Exploratory risk stratification by predefined COGAS cutoffs for operationally defined 6-month delayed neuropsychiatric sequelae.

Cutoff	Unadjusted OR (95% CI)	Adjusted OR (95% CI)	Sensitivity	Specificity	PPV	NPV
COGAS ≥ 2	3.02 (1.01–9.00)	2.37 (0.61–9.24)	57.1%	69.4%	9.2%	96.8%
COGAS ≥ 3	3.16 (0.93–10.72)	2.02 (0.45–9.16)	28.6%	88.8%	12.1%	95.8%
COGAS ≥ 4	21.33 (2.76–164.67)	11.19 (1.03–122.09)	14.3%	99.2%	50.0%	95.5%

Note. Odds ratios are shown with 95% confidence intervals. The adjusted model included initial GDS, troponin I, and CO exposure duration. Sensitivity, specificity, PPV, and NPV were calculated for each predefined COGAS cutoff. CO, carbon monoxide; DNS, delayed neuropsychiatric sequelae; GDS, Global Deterioration Scale; NPV, negative predictive value; OR, odds ratio; PPV, positive predictive value.

## Data Availability

The study dataset cannot be made publicly available because of ethical and legal constraints. Qualified researchers may contact the corresponding author with a justified request for data access. De-identified data may be provided only after approval by the relevant Institutional Review Board.

## Supplementary Materials

The following supporting information can be downloaded at https://www.mdpi.com/article/10.3390/jcm15114322/s1: Table S1: Global Deterioration Scale categories; Table S2: Baseline characteristics according to the availability of initial GDS assessment; Table S3: Components and trajectories of the primary operationally defined 6-month delayed neuropsychiatric sequelae outcome; Table S4: Sensitivity analysis using alternative outcome definitions for delayed neuropsychiatric sequelae; Table S5: Component-specific sensitivity analysis of the association between the COGAS score and DNS progression from 1 to 6 months; Table S6: Sensitivity analysis using the 4-component COGAS score excluding the HBOT item; Table S7: Associations between predefined COGAS cutoffs and operationally defined 6-month delayed neuropsychiatric sequelae across different adjustment models; Figure S1: Discrimination performance of the COGAS score for operationally defined 6-month DNS.

## Abbreviations

The following abbreviations are used in this manuscript:

PR-AUC	Area under the precision-recall curve
ROC-AUC	Area under the receiver operating characteristic curve
CO	Carbon monoxide
CARE CO	Carbon Monoxide Intoxication in Korea: Prospective Cohort
COGAS	Carbon monoxide-associated global assessment score
CO-Hb	Carboxyhemoglobin
CI	Confidence interval
DNS	Delayed neuropsychiatric sequelae
ED	Emergency department
GCS	Glasgow Coma Scale
GDS	Global Deterioration Scale
HBOT	Hyperbaric oxygen therapy
IRB	Institutional Review Board
NPV	Negative predictive value
OR	Odds ratio
PPV	Positive predictive value

## References

[1] Shin M., Bronstein A.C., Glidden E., Malone M., Chang A., Law R., Boehmer T.K., Strosnider H., Yip F. (2023). Morbidity and mortality of unintentional carbon monoxide poisoning: United States 2005 to 2018. Ann. Emerg. Med..

[2] Hampson N.B. (2023). Carbon monoxide poisoning mortality in the United States from 2015–2021. Clin. Toxicol..

[3] Mattiuzzi C., Lippi G. (2020). Worldwide epidemiology of carbon monoxide poisoning. Hum. Exp. Toxicol..

[4] Choi S. (1983). Delayed neurologic sequelae in carbon monoxide intoxication. Arch. Neurol..

[5] Hampson N.B., Piantadosi C.A., Thom S.R., Weaver L.K. (2012). Practice recommendations in the diagnosis, management, and prevention of carbon monoxide poisoning. Am. J. Respir. Crit. Care Med..

[6] Min S.K. (1986). A brain syndrome associated with delayed neuropsychiatric sequelae following acute carbon monoxide intoxication. Acta Psychiatr. Scand..

[7] Thorn S.R., Keim L.W. (1989). Carbon monoxide poisoning: A review epidemiology, pathophysiology, clinical findings, and treatment options including hyperbaric oxygen therapy. J. Toxicol. Clin. Toxicol..

[8] Thom S.R., Taber R.L., Mendiguren I.I., Clark J.M., Hardy K.R., Fisher A.B. (1995). Delayed neuropsychologic sequelae after carbon monoxide poisoning: Prevention by treatment with hyperbaric oxygen. Ann. Emerg. Med..

[9] Pepe G., Castelli M., Nazerian P., Vanni S., Del Panta M., Gambassi F., Botti P., Missanelli A., Grifoni S. (2011). Delayed neuropsychological sequelae after carbon monoxide poisoning: Predictive risk factors in the Emergency Department. A retrospective study. Scand. J. Trauma Resusc. Emerg. Med..

[10] Namgung M., Oh J., Ahn C., Kim C.W., Lee H., Kang H. (2022). Association between Glasgow Coma Scale in early carbon monoxide poisoning and development of delayed neurological sequelae: A meta-analysis. J. Pers. Med..

[11] Lee H., Kang H., Ko B.S., Oh J., Lim T.H., Cho Y. (2021). Initial creatine kinase level as predictor for delayed neuropsychiatric sequelae associated with acute carbon monoxide poisoning. Am. J. Emerg. Med..

[12] Cha Y.S., Kim H., Do H.H., Kim H.I., Kim O.H., Cha K.C., Lee K., Hwang S. (2018). Serum neuron-specific enolase as an early predictor of delayed neuropsychiatric sequelae in patients with acute carbon monoxide poisoning. Hum. Exp. Toxicol..

[13] Zhang Y., Gao N., Wang Y., Hu W., Wang Z., Pang L. (2024). Association between serum neuron-specific enolase at admission and the risk of delayed neuropsychiatric sequelae in adults with carbon monoxide poisoning: A meta-analysis. Biomol. Biomed..

[14] Jeon S.B., Sohn C.H., Seo D.W., Oh B.J., Lim K.S., Kang D.W., Kim W.Y. (2018). Acute brain lesions on magnetic resonance imaging and delayed neurological sequelae in carbon monoxide poisoning. JAMA Neurol..

[15] Ahn C., Oh J., Kim C.W., Lee H., Lim T.H., Kang H. (2022). Early neuroimaging and delayed neurological sequelae in carbon monoxide poisoning: A systematic review and meta-analysis. Sci. Rep..

[16] Pan K.T., Shen C.H., Lin F.G., Chou Y.C., Croxford B., Leonardi G., Huang K.L. (2019). Prognostic factors of carbon monoxide poisoning in Taiwan: A retrospective observational study. BMJ Open.

[17] Zhang Y., Lu Q., Jia J., Xiang D., Xi Y. (2021). Multicenter retrospective analysis of the risk factors for delayed neurological sequelae after acute carbon monoxide poisoning. Am. J. Emerg. Med..

[18] Sert E.T., Kokulu K., Mutlu H. (2021). Clinical predictors of delayed neurological sequelae in charcoal-burning carbon monoxide poisoning. Am. J. Emerg. Med..

[19] Han S., Choi S., Nah S., Lee S.U., Cho Y.S., Kim G.W., Lee Y.H. (2021). Cox regression model of prognostic factors for delayed neuropsychiatric sequelae in patients with acute carbon monoxide poisoning: A prospective observational study. Neurotoxicology.

[20] Weaver L.K., Valentine K.J., Hopkins R.O. (2007). Carbon monoxide poisoning: Risk factors for cognitive sequelae and the role of hyperbaric oxygen. Am. J. Respir. Crit. Care Med..

[21] Afzal M., Agarwal S., Elshaikh R.H., Babker A.M.A., Choudhary R.K., Prabhakar P.K., Zahir F., Sah A.K. (2025). Carbon monoxide poisoning: Diagnosis, prognostic factors, treatment strategies, and future perspectives. Diagnostics.

[22] Weaver L.K., Hopkins R.O., Chan K.J., Churchill S., Elliott C.G., Clemmer T.P., Orme J.F., Thomas F.O., Morris A.H. (2002). Hyperbaric oxygen for acute carbon monoxide poisoning. N. Engl. J. Med..

[23] Lin C.H., Su W.H., Chen Y.C., Feng P.H., Shen W.C., Ong J.R., Wu M.Y., Wong C.S. (2018). Treatment with normobaric or hyperbaric oxygen and its effect on neuropsychometric dysfunction after carbon monoxide poisoning: A systematic review and meta-analysis of randomized controlled trials. Medicine.

[24] Kim S.H., Lee Y., Kang S., Paik J.H., Kim H., Cha Y.S. (2022). Derivation and validation of a score for predicting poor neurocognitive outcomes in acute carbon monoxide poisoning. JAMA Netw. Open.

[25] Reisberg B., Ferris S.H., de Leon M.J., Crook T. (1982). The Global Deterioration Scale for assessment of primary degenerative dementia. Am. J. Psychiatry.

[26] Kim S.J., Thom S.R., Kim H., Hwang S.O., Lee Y., Park E.J., Lee S.J., Cha Y.S. (2020). Effects of Adjunctive Therapeutic Hypothermia Combined with Hyperbaric Oxygen Therapy in Acute Severe Carbon Monoxide Poisoning. Crit. Care Med..

[27] Fujita M., Todani M., Kaneda K., Suzuki S., Wakai S., Kikuta S., Sasaki S., Hattori N., Yagishita K., Kuwata K. (2021). Use of hyperbaric oxygen therapy for preventing delayed neurological sequelae in patients with carbon monoxide poisoning: A multicenter, prospective, observational study in Japan. PLoS ONE.

[28] Gao X., Wei W., Yang G.D. (2024). Clinical factors for delayed neuropsychiatric sequelae from acute carbon monoxide poisoning: A retrospective study. Front. Med..

[29] Nah S., Choi S., Kim G.W., Moon J.E., Lee Y.H., Han S. (2021). Prediction of delayed neuropsychiatric sequelae after carbon monoxide poisoning via serial determination of serum neuron-specific enolase levels. Hum. Exp. Toxicol..

