# Supplementary material for: Association Between the COGAS Score and Delayed Neuropsychiatric Sequelae After Acute Carbon Monoxide Poisoning
Source: J Clin Med. 2026 Jun 3;15(11):4322. doi: 10.3390/jcm15114322 (PMC13257806; doi:10.3390/jcm15114322)
Supplement: Supplementary file 1 [file jcm-15-04322-s001.zip › jcm-4323623-supplementary.pdf]

Supplementary Materials

Table S1. Global Deterioration Scale categories.

Stage	Cognitive dysfunction	Clinical characteristics
1	No cognitive decline	● Clinically within normal limits<br>● No subjective memory complaints<br>● No objective memory impairment identified during clinical interview
2	Very mild cognitive decline	● Subjective complaints of mild memory difficulty<br>● Common examples include misplacing familiar belongings and difficulty recalling the names of well-known individuals<br>● No objective memory impairment is evident during clinical interview<br>● No clear functional limitation is observed in occupational or social settings<br>● Patients generally show appropriate awareness of these changes
3	Mild cognitive decline	● Earliest clearly discernible cognitive impairment<br>● Objective memory deficits may be detectable only on detailed evaluation by an experienced clinician, and impaired concentration may be observed on formal testing<br>● Patients may have difficulty recalling names after new introductions or retaining information from recently read material<br>● Subtle decline may become apparent in more demanding occupational or social settings, including reduced work

		performance, noticeable word-finding difficulty, misplacement of valuable items, or disorientation in unfamiliar places<br>● Symptoms are often minimized or denied, and mild to moderate anxiety may accompany increasing difficulty with complex daily demands
4	Moderate cognitive decline	● Definite cognitive impairment is apparent on careful clinical interview<br>● Deficits may involve impaired concentration, reduced awareness of recent or current events, impaired recall of personal historical information, and decreased ability to travel independently or manage finances<br>● Complex tasks can no longer be carried out accurately and efficiently<br>● Basic orientation to time and persons is generally maintained, familiar individuals can still be distinguished from unfamiliar people, and travel to well-known places is usually preserved<br>● Denial is common, and patients may show blunted affect and avoidance of previously demanding situations
5	Moderately severe cognitive decline	● Patients are no longer able to maintain independent living without some degree of assistance<br>● On interview, they may be unable to recall major details of their current lives, such as their address, telephone number, names of close relatives, or the school from which they graduated<br>● Partial disorientation to time or place may be present

		• In highly educated individuals, impaired performance on backward counting tasks may be evident<br>• Core personal identity is usually preserved, including their own name and generally the names of a spouse and children<br>• Basic functions such as eating and toileting are typically maintained, although difficulty selecting appropriate clothing or dressing correctly may occur
6	Severe cognitive decline	• Patients may at times fail to recall the name of a spouse or primary caregiver, despite being highly dependent on that person for daily survival<br>• Awareness of recent events and current experiences is markedly impaired<br>• Remote memory may be partially retained, although it is often fragmentary or unreliable<br>• Patients are frequently disoriented to surroundings, year, or season, and may have difficulty performing simple counting tasks<br>• Considerable assistance is required for activities of daily living<br>• Associated behavioral and neuropsychiatric features may include delusional thinking, repetitive or compulsive behaviors, anxiety, agitation, uncharacteristic aggression, and marked reduction in goal-directed thought or initiative
7	Very severe cognitive decline	• Verbal communication is essentially absent<br>• In many cases, no meaningful speech remains, with only nonverbal vocalizations such as grunting preserved

	• Patients are incontinent and require assistance with feeding and toileting<br>• Psychomotor abilities are profoundly impaired, including loss of independent ambulation<br>• Widespread cortical dysfunction with focal neurologic signs may be present

Table S2. Baseline characteristics according to the availability of initial GDS assessment.

	Initial GDS available (n = 314)	Initial GDS unavailable (n = 1440)	*p*-value
Age	52 (36–62)	45 (34–60)	0.013
Sex			0.735
Woman	122 (38.9)	542 (37.6)	
Man	192 (61.1)	898 (62.4)	
Intentionality	119 (37.9)	625 (43.6)	0.076
CO source			0.008
Charcoal	233 (74.2)	1,024 (71.2)	
Firewood	16 (5.1)	72 (5.0)	
Gas	45 (14.3)	154 (10.7)	
Fire	20 (6.4)	181 (12.6)	
Other	0 (0.0)	8 (0.6)	
Drug co-ingestion	18 (5.7)	178 (12.4)	<0.001
GCS score	15 (12–15)	15 (12–15)	0.058
Comorbidities			
Diabetes mellitus	37 (11.8)	156 (10.8)	0.701
Hypertension	70 (22.3)	265 (18.4)	0.133
Cardiovascular disease	7 (2.2)	24 (1.7)	0.653
Psychiatric disease	29 (9.2)	224 (15.6)	0.005
Alcohol co-ingestion	21 (6.7)	153 (10.9)	0.032
Current smoker	129 (41.1)	592 (42.2)	0.758
Symptoms and signs at ED presentation			
Loss of consciousness	170 (54.1)	776 (54.0)	>0.999
Shock	6 (1.9)	53 (3.7)	0.161
Seizure	4 (1.3)	17 (1.2)	>0.999
HBOT treatment	306 (97.5)	1,165 (80.9)	<0.001
CO exposure duration (hours)	4.25 (1.50–8.00)	3.00 (1.00–8.00)	0.006
Laboratory findings			
CO-Hb (%)	23.15 (11.40–32.30)	15.10 (5.70–27.40)	<0.001
Bicarbonate (mmol/L)	20.90 (19.00–22.60)	21.90 (19.30–23.80)	<0.001
Lactate (mmol/L)	2.07 (1.27–3.29)	2.18 (1.43–3.50)	0.064
Creatinine (mg/dL)	0.81 (0.66–0.99)	0.81 (0.68–1.00)	0.370
Creatine kinase (U/L)	142.50 (90.00–369.00)	129.00 (84.00–252.00)	0.024
Troponin I (pg/mL)	15.50 (15.00–189.00)	15.00 (6.00–126.99)	<0.001
COGAS score	1 (0–2)	1 (0–2)	0.912

Note. Data are presented as n (%) or median (interquartile range), as appropriate. Troponin I values were harmonized to pg/mL; low values reflect institutional reporting conventions during the study period. CO, carbon monoxide; CO-Hb, carboxyhemoglobin; ED, emergency department; GCS, Glasgow Coma Scale; GDS, Global Deterioration Scale; HBOT, hyperbaric oxygen therapy.

Table S3. Components and trajectories of the primary operationally defined 6-month delayed neuropsychiatric sequelae outcome.

Component	Definition	Detailed GDS trajectory	n (%)
Classical DNS-like deterioration	Initial GDS 1–3 followed by 6-month GDS 4–7	Initial GDS 1–3 → 6-month GDS 4–7	9 (64.3)
Progressive deterioration	Initial GDS ≥4 with ≥1-point worsening at 6 months	Initial GDS 4 → 6-month GDS 7	2 (14.3)
		Initial GDS 5 → 6-month GDS 7	2 (14.3)
		Initial GDS 6 → 6-month GDS 7	1 (7.1)
Operationally defined 6-month DNS	Either of the above criteria		14 (100.0)

Note. The two outcome components were mutually exclusive. All five patients with progressive deterioration worsened to a 6-month GDS of 7. DNS, delayed neuropsychiatric sequelae; GDS, Global Deterioration Scale.

Table S4. Sensitivity analysis using alternative outcome definitions for delayed neuropsychiatric sequelae.

DNS definition	Method	Events	N	Unadjusted model		Adjusted model	
				OR (95% CI)	*p*-value	OR (95% CI)	*p*-value
Classical DNS-like deterioration	Conventional	9	272	1.57 (0.83–2.91)	0.149	2.51 (1.11–5.70)	0.024
	Firth	9	272	1.58 (0.85–2.86)	0.142	2.39 (1.08–5.23)	0.032
Progressive deterioration	Conventional	5	272	3.61 (1.55–10.18)	0.006	2.07 (0.70–7.02)	0.204
	Firth	5	272	3.33 (1.50–8.69)	0.003	1.87 (0.70–5.52)	0.211
Operationally defined 6-month DNS	Conventional	14	272	2.16 (1.30–3.65)	0.003	2.19 (1.14–4.35)	0.020
	Firth	14	272	2.13 (1.30–3.55)	0.003	2.09 (1.11–4.06)	0.022

Note. Odds ratios are expressed per 1-point increase in the COGAS score. The adjusted model included initial GDS, troponin I, and CO exposure duration. DNS definitions are shown in Supplementary Table S3. CI, confidence interval; CO, carbon monoxide; DNS, delayed neuropsychiatric sequelae; GDS, Global Deterioration Scale; OR, odds ratio.

Table S5. Component-specific sensitivity analysis of the association between the COGAS score and DNS progression from 1 to 6 months.

DNS progression from 1 to 6 months	Method	Events	N	Unadjusted model		Adjusted model	
				OR (95% CI)	*p*-value	OR (95% CI)	*p*-value
Classical DNS-like deterioration	Conventional	3	272	2.05 (0.71–6.15)	0.174	4.00 (1.12–15.21)	0.027
	Firth	3	272	2.01 (0.75–5.51)	0.158	3.18 (0.91–10.35)	0.070
Progressive deterioration	Conventional	2	272	3.14 (0.87–15.31)	0.095	1.75 (0.25–16.08)	0.583
	Firth	2	272	2.80 (0.89–11.21)	0.078	1.47 (0.28–7.70)	0.616
Operationally defined 6-month DNS	Conventional	5	272	2.45 (1.09–5.97)	0.033	2.70 (0.92–8.06)	0.067
	Firth	5	272	2.37 (1.10–5.46)	0.029	2.40 (0.85–6.78)	0.097

Note. Odds ratios are expressed per 1-point increase in the COGAS score. The adjusted model included initial GDS, troponin I, and CO exposure duration. DNS progression was assessed from 1-month to 6-month GDS. CI, confidence interval; CO, carbon monoxide; DNS, delayed neuropsychiatric sequelae; GDS, Global Deterioration Scale; OR, odds ratio.

Table S6. Sensitivity analysis using the 4-component COGAS score excluding the HBOT item.

Score definition	Method	Events	N	Unadjusted model		Adjusted model	
				OR (95% CI)	*p*-value	OR (95% CI)	*p*-value
4-component COGAS, excluding HBOT	Conventional	14	272	2.31 (1.31–4.25)	0.005	2.10 (0.91–4.98)	0.083
	Firth	14	272	2.27 (1.30–4.11)	0.004	2.02 (0.91–4.66)	0.086

Note. Odds ratios are expressed per 1-point increase in the 4-component COGAS score excluding the HBOT item. The adjusted model included initial GDS, troponin I, and CO exposure duration. CI, confidence interval; CO, carbon monoxide; GDS, Global Deterioration Scale; HBOT, hyperbaric oxygen therapy; OR, odds ratio.

Table S7. Associations between predefined COGAS cutoffs and operationally defined 6-month delayed neuropsychiatric sequelae across different adjustment models.

**Model**	**Method**	COGAS ≥2	COGAS ≥3	COGAS ≥4
Univariable	Conventional	3.02 (1.01–9.00) p=0.047	3.16 (0.93–10.72) p=0.065	21.33 (2.76–164.67) p=0.003
	Firth	2.95 (1.03–8.85) p=0.044	3.33 (0.94–10.24) p=0.062	20.52 (2.95–143.75) p=0.004
Adjusted for initial GDS	Conventional	2.80 (0.77–10.22) p=0.118	2.57 (0.60–10.99) p=0.202	16.90 (1.99–143.42) p=0.010
	Firth	2.77 (0.77–9.80) p=0.118	2.62 (0.61–10.44) p=0.189	15.34 (2.03–115.16) p=0.011
Adjusted for CO exposure duration	Conventional	2.88 (0.91–9.08) p=0.072	2.90 (0.77–10.94) p=0.116	19.43 (2.48–152.26) p=0.005
	Firth	2.80 (0.92–8.85) p=0.070	3.00 (0.77–10.37) p=0.107	17.89 (2.55–126.45) p=0.006
Adjusted for troponin I	Conventional	2.38 (0.74–7.63) p=0.144	2.28 (0.60–8.68) p=0.227	12.44 (1.15–135.06) p=0.038
	Firth	2.37 (0.76–7.46) p=0.135	2.42 (0.60–8.09) p=0.199	11.45 (1.04–100.01), p=0.047
Adjusted for initial GDS and CO exposure duration	Conventional	2.71 (0.72–10.18) p=0.141	2.43 (0.54–10.98) p=0.247	16.42 (1.93–139.42) p=0.010
	Firth	2.65 (0.71–9.67) p=0.142	2.43 (0.54–10.27) p=0.240	14.32 (1.90–106.98) p=0.012
Adjusted for initial GDS and troponin I	Conventional	2.40 (0.64–9.08) p=0.196	2.08 (0.48–9.14) p=0.331	11.22 (1.01–124.55) p=0.049
	Firth	2.41 (0.64–8.70) p=0.188	2.12 (0.49–8.44) p=0.302	9.72 (0.92–85.25) p=0.057
Fully adjusted	Conventional	2.37 (0.61–9.24) p=0.214	2.02 (0.45–9.16) p=0.360	11.19 (1.03–122.09) p=0.048
	Firth	2.36 (0.61–8.76) p=0.208	2.03 (0.46–8.33) p=0.336	9.38 (0.93–80.36) p=0.057

Note. Values are ORs with 95% CIs for each predefined COGAS cutoff. Each cutoff was modeled separately, comparing patients at or above the cutoff with those below it. All models included 272 patients and 14 outcome events. Adjusted models included the covariates indicated in the model column. CI, confidence interval; CO, carbon monoxide; DNS, delayed neuropsychiatric sequelae; GDS, Global Deterioration Scale; OR, odds ratio.

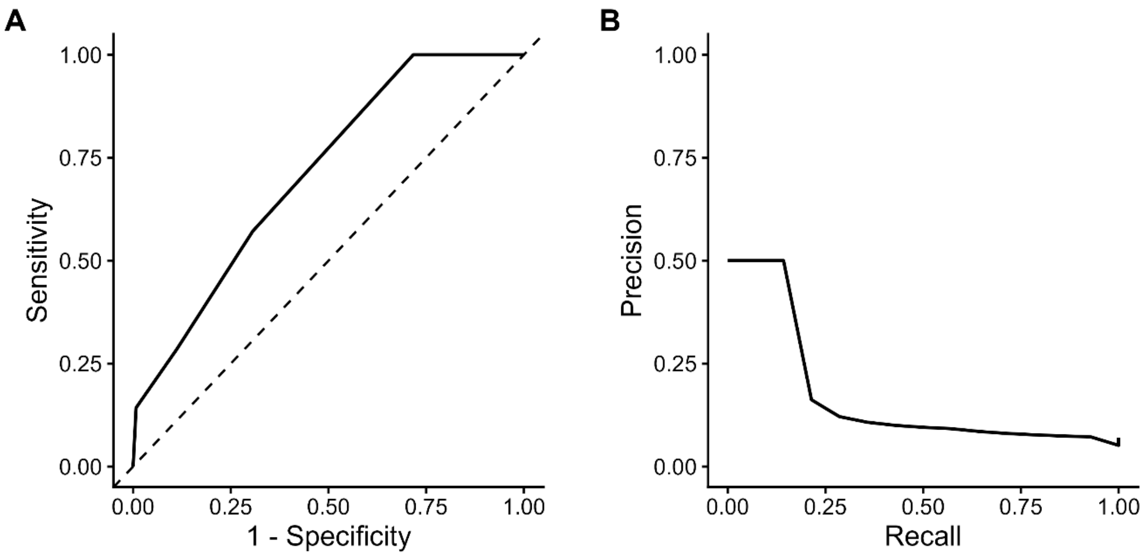

Figure S1. Discrimination performance of the COGAS score for operationally defined 6-month DNS. (A) Receiver operating characteristic curve for the COGAS score. The ROC-AUC was 0.712 (95% CI, 0.596–0.828). (B) Precision-recall curve for the COGAS score. The PR-AUC was 0.162. These analyses were exploratory because of the limited number of outcome events. DNS, delayed neuropsychiatric sequelae; ROC-AUC, area under the receiver operating characteristic curve; PR-AUC, area under the precision-recall curve.
